# A herbal formula comprising Rosae Multiflorae Fructus and Lonicerae Japonicae Flos, attenuates collagen-induced arthritis and inhibits TLR4 signalling in rats

**DOI:** 10.1038/srep20042

**Published:** 2016-02-10

**Authors:** Brian Chi Yan Cheng, Hua Yu, Hui Guo, Tao Su, Xiu-Qiong Fu, Ting Li, Hui-Hui Cao, Anfernee Kai-Wing Tse, Zheng-Zhi Wu, Hiu-Yee Kwan, Zhi-Ling Yu

**Affiliations:** 1 Consun Chinese Medicines Research Centre for Renal Diseases, Hong Kong Baptist University, Hong Kong; 2 Centre for Cancer and Inflammation Research, School of Chinese Medicine, Hong Kong Baptist University, Hong Kong; 3 Institute of Integrated Bioinfomedicine & Translational Science, HKBU Shenzhen Research Institute and Continuing Education, Shenzhen, PRC; 4 Shenzhen Institute of Geriatrics, Shenzhen, PRC

## Abstract

RL, a traditional remedy for Rheumatoid arthritis (RA), comprises two edible herbs, Rosae Multiflorae Fructus and Lonicerae Japonicae Flos. We have reported that RL could inhibit the production of inflammatory mediators in immune cells. Here we investigated the effects and the mechanism of action of RL in collagen-induced arthritis (CIA) rats. RL significantly increased food intake and weight gain of CIA rats without any observable adverse effect; ameliorated joint erythema and swelling; inhibited immune cell infiltration, bone erosion and osteophyte formation in joints; reduced joint protein expression levels of TLR4, phospho-TAK1, phospho-NF-κB p65, phospho-c-Jun and phospho-IRF3; lowered levels of inflammatory factors (TNF-α, IL-6, IL-1β, IL-17A and MCP-1 in sera and TNF-α, IL-6, IL-1β and IL-17A in joints); elevated serum IL-10 level; reinvigorated activities of antioxidant SOD, CAT and GSH-Px in the liver and serum; reduced Th17 cell proportions in splenocytes; inhibited splenocyte proliferation and activation; and lowered serum IgG level. In conclusion, RL at nontoxic doses inhibited TLR4 signaling and potently improved clinical conditions of CIA rats. These findings provide further pharmacological justifications for the traditional use of RL in RA management.

Rheumatoid arthritis (RA), a chronic autoimmune disease, is characterized by synovitis that drives extracellular matrix degradation and consequent cartilage damage and bone erosion. Prevalent in about 1% of the world population, RA ruins the quality of life of patients^1^. Conventional drug treatment options do not provide satisfactory efficacy and even cause serious adverse reactions^2^. Safe and effective novel targeted therapeutic agents are desperately needed. Researchers are seeking new approaches based on understanding RA pathology. Molecular evidence indicates that toll-like receptors (TLRs), especially TLR4, play important pathogenic roles in RA^3^. Increased expression of TLR4 has been observed in cells from human RA joints^4^, where both exogenous and endogenous TLR4 ligands have been detected^5^. TLR4 initiates signals for different pathways that activate transcription factors like nuclear factor-κB (NF-κB), activator protein-1 (AP-1) and interferon regulatory factor 3 (IRF3), leading to the production of inflammatory cytokines, chemokines and tissue-destructive enzymes in synovium^3^. Activation of TLR4 signalling promotes the differentiation of CD4^+^ T cells into pathogenic Th17 effectors, driving cartilage and bone erosion^6^,^7^,^8^,^9^. TLR4 signalling also plays a role in RA by inducing auto-antigen-specific adaptive immune responses^10^, thereby resulting in persistent joint damage^3^. Attenuating TLR4 signalling pathways is believed to be beneficial in RA management^11^,^12^.

Multi-target Chinese medicines with low toxicity and high efficacy are alternative options complementary to the conventional RA drugs^13^. Rosae Multiflorae Fructus (dried fruits of *Rosa multiflora, Yingshi* in Chinese) and Lonicerae Japonicae Flos (dried flowers of *Lonicera japonica, Jinyinhua* in Chinese) have traditionally been prescribed by Chinese medicine practitioners for treating various inflammatory disorders including RA^14^,^15^,^16^. Extracts/constituents of these two herbs attenuate animal arthritis and inhibit TLR4 signalling^17^,^18^,^19^,^20^,^21^,^22^. We have previously reported that RL, a herbal formula composed of these two herbs, could inhibit various components of the TLR4 signalling pathways^23^,^24^. Therefore, here we examined if RL could improve clinical conditions in collagen-induced arthritis (CIA) rats, a model that is well established for studying human RA, and investigated the involvement of TLR4 signalling in the effects of RL.

## Results

### RL improved clinical arthritic conditions in CIA rats

**Figure 1:**
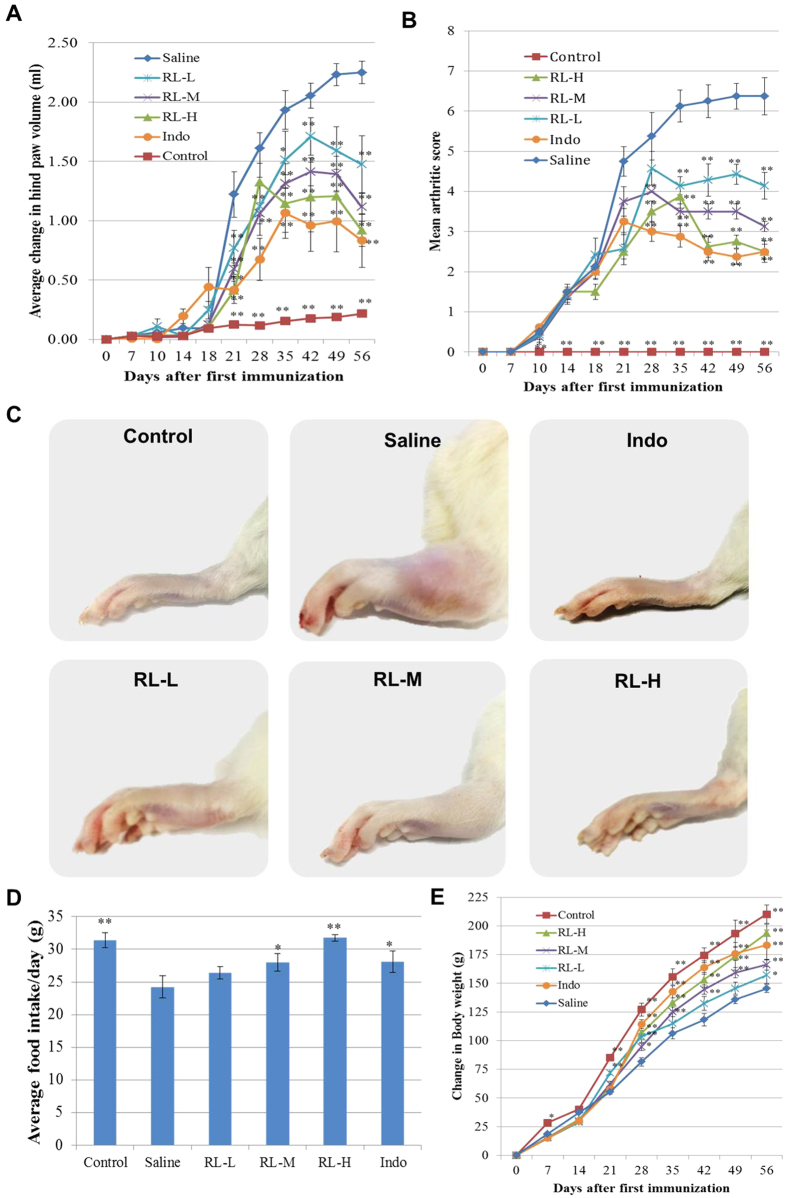
Effects of RL on disease progression in CIA rats. Male Wistar rats were immunized on day 0 and day 7 with bovine type II collagen for CIA or with vehicle. CIA rats were intragastrically (i.g.) given vehicle (saline), indomethacin (Indo), low dose of RL (RL-L), middle dose of RL (RL-M) or high dose of RL (RL-H) (n = 8 for each group) daily from days 14 to 56. (**A**) Average volumes of hind paws. (**B**) Mean arthritic score. (**C**) Representative photographs showing the gross features of hind paws at day 57. (**D**) Average daily food intake during the experimental period. (**E**) Changes in body weight over the experimental period. Values are the mean ± SEM (n = 8). *P < 0.05, **P < 0.01 compared with the immunized control (saline).

In the present study, we investigated the *in vivo* efficacy of RL in CIA rats. Rats immunized with bovine type II collagen began to develop arthritis in the first week. The initial manifestation of arthritis was erythema and swelling of ankle joints, followed by the inflammation of the metatarsal and inter-phalangeal joints. Disease progression can be evaluated by measuring paw swelling volume, which is an indicator of the degree of inflammation. In order to evaluate the anti-arthritic efficacy of RL, the paw volume changes were quantified using plethysmometry. At all three doses, RL significantly ameliorated paw swelling. At the end of the experiment, more significant reductions of paw volume were observed in groups treated with RL (660 mg/kg) and indomethacin (2.5 mg/kg), a nonsteroidal anti-inflammatory drug (NSAID) used as the positive control (Fig. 1A).

Overall arthritis severity in CIA rats is commonly assessed by macroscopic clinical scoring. Beginning from day 7, the arthritis score of CIA rats increased progressively and reached about 6.5 in the last week. Administration of RL significantly attenuated all clinical signs of arthritis indicated by the significant reduction in the mean arthritis score (Fig. 1B). Representative photographs showing morphological changes of hind paws of rats from different groups on day 57 are shown in Fig. 1C.

Beginning from day 21, CIA rats gained significantly less weight than the normal rats. The average food intake of CIA rats was significantly reduced. RL effectively improved the food intake and ameliorated weight loss caused by CIA. Moreover, no observable adverse reaction was found in RL-treated groups during the entire experimental period. (Fig. 1D,E).

### RL attenuated the radiographic damage of CIA rats

**Figure 2:**
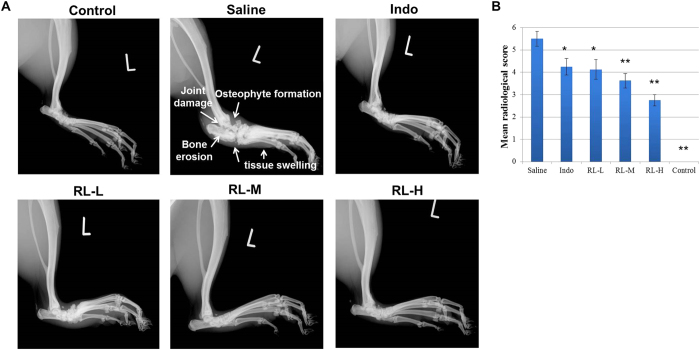
Effects of RL on the radiographs of CIA rats. (**A**) Representative radiographs of the hind limbs showing the talocrural joints at day 57. (**B**) Mean radiological score of the radiographs. Values are the mean ± SEM (n = 8). *P < 0.05, **P < 0.01 compared with the immunized control (saline).

Radiographic examination of the talocrural joints revealed tissue swelling, bone matrix resorption, osteophyte formation and bone erosion at the joint margins in CIA rats. No pathogenic change was observed in normal rats. RL markedly ameliorated soft tissue swelling and the degree of joint damage (Fig. 2A). Significant reduction in radiographic score was observed in RL-treated groups (Fig. 2B).

### RL improved the histological parameters of CIA rats

**Figure 3:**
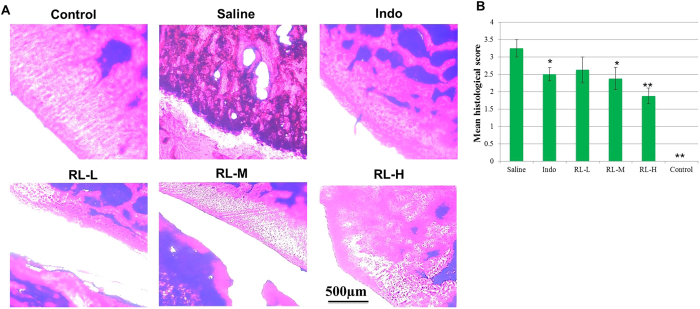
Effects of RL on histological parameters in the talocrural joints of CIA rats. (**A**) Representative histological observation from light microscope of the talocrural joint sections stained with H&E for inflammatory cell influx and bone destruction (magnification ×100). (**B**) Mean score of the histological observation. Values are the mean ± SEM (n = 8). *P < 0.05, **P < 0.01 compared with the immunized control (saline).

In order to determine whether RL prevented articular destruction, talocrural joints were analysed histologically. Histological examinations showed massive infiltration of inflammatory cells, pannus invasion, cartilage damage, and subchondral bone erosion in CIA rats (Fig. 3A). Histological scores of individual groups were shown in (Fig. 3B).

### RL inhibited TLR4 signalling in CIA rats

**Figure 4:**
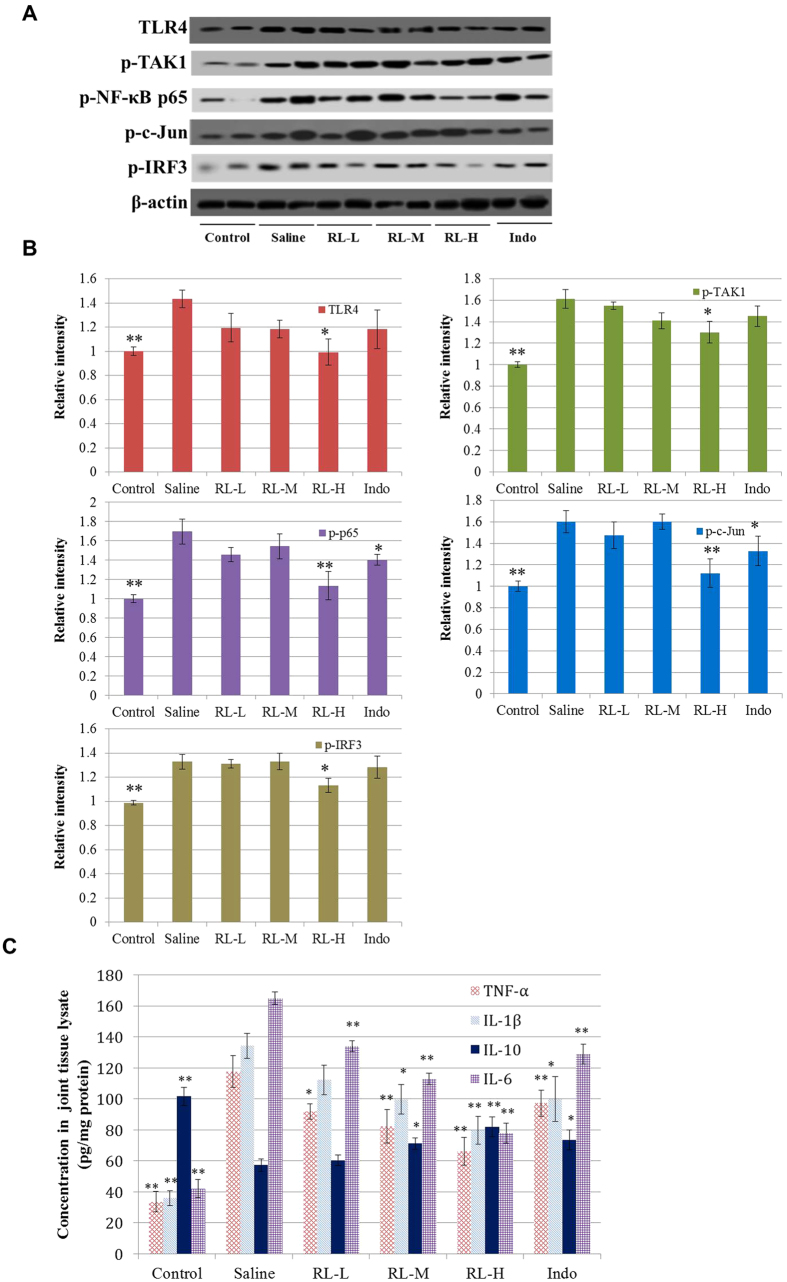
Effects of RL on TLR4 signalling in joint tissues of CIA rats. (**A**) Representative western blots showing the protein expression of TLR4, phospho-TAK1, phospho-NF-κB p65, phospho-c-Jun, phospho-IRF3 and β-actin (as loading control) in joint tissues. (**B**) Relative intensity values from band densitometry. The values are the mean ± SEM of the band density normalized to the loading control in relation to mean value of untreated control (control) normalized to the loading control (n = 8). (**C**) Levels of cytokines from joint tissue lysates determined by Milliplex MAP Rat Cytokine/Chemokine Panel or ELISA at the end of the experiment. Values are the mean ± SEM (n = 8). *P < 0.05, **P < 0.01 compared with the immunized control (saline).

**Figure 5:**
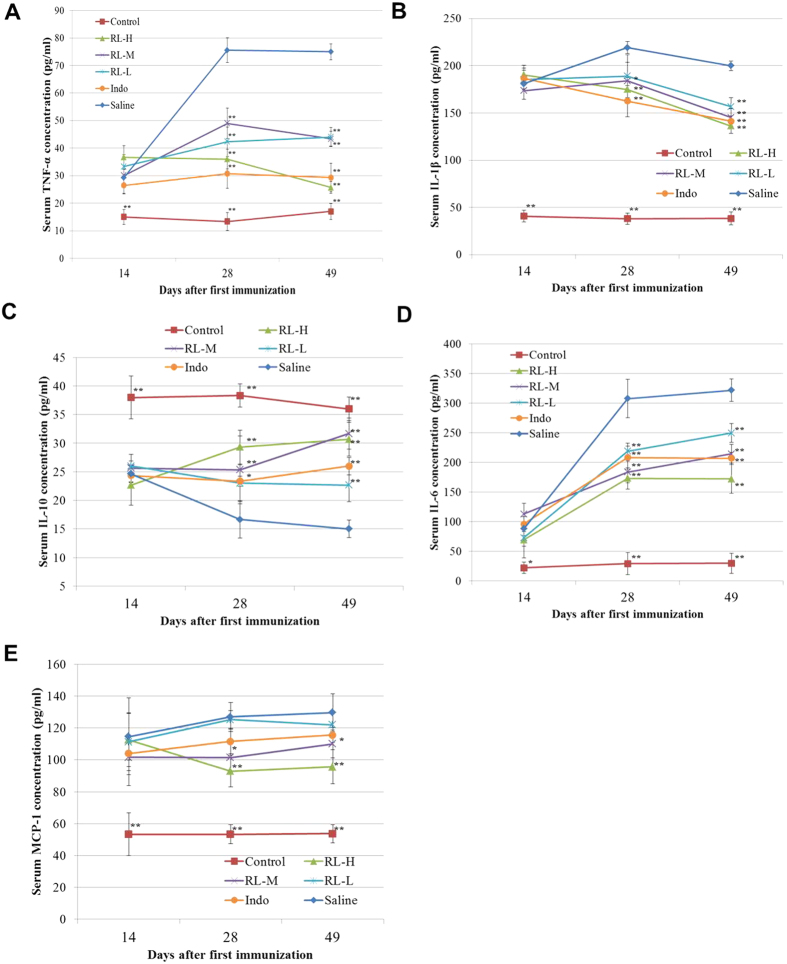
Effects of RL on serum levels of cytokines and chemokines in CIA rats. Serum levels of cytokines and chemokines were determined by Milliplex MAP Rat Cytokine/Chemokine Panel or ELISA at different time points. (**A**) Changes in serum TNF-α levels. (**B**) Changes in serum IL-1β levels. (**C**) Changes in serum IL-10 levels. (**D**) Changes in serum IL-6 levels. (**E**) Changes in serum MCP-1 levels. Values are the mean ± SEM (n = 8). *P < 0.05, **P < 0.01 compared with the immunized control (saline).

In a previous study, we have demonstrated that RL could inhibit the TAK1/NF-κB, TAK1/AP-1, TBK1/IRF3 pathways in LPS-stimulated murine RAW 264.7 and human THP-1 cells. Increased expression/activities of these TLR4 signalling components (TAK1, NF-κB, AP-1 and IRF3) in CIA rats demonstrated their involvement in the pathology of CIA. RL reduced the upregulated protein levels of TLR4, phospho-TAK1, phospho-NF-κB p65, phospho-c-Jun and phospho-IRF3 in CIA joint tissues (Fig. 4A,B). Upon ligand binding, TLR4 recruits several adaptors which lead to activation of TAK1. Transcription of NF-κB, AP-1 and IRF3 initiates the production of pro-inflammatory cytokines and effector cytokines such as TNF-α, IL-1β and IL-6, which contribute to the pathogenesis of CIA. RL mitigated the upregulated levels of pro-inflammatory TNF-α, IL-1β and IL-6, while elevated the levels of anti-inflammatory IL-10 in both sera and joint tissue lysates (Figs 4C and 5A–D). RL also lowered the level of MCP-1 in sera (Fig. 5E).

### RL rejuvenated the declined activities of endogenous antioxidant enzymes in CIA rats

**Figure 6:**
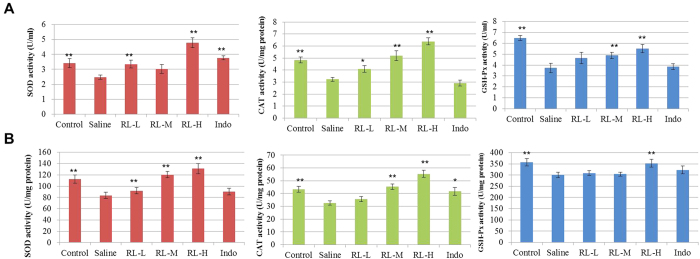
Effects of RL on the activities of antioxidant enzymes in CIA. (**A**) Antioxidant enzyme (SOD, CAT, and GSH-Px) activities in sera collected on day 49 after first immunization. (**B**) Antioxidant enzyme (SOD, CAT, and GSH-Px) activities in homogenized liver tissues at the end of the experiment. Values are the mean ± SEM (n = 8). *P < 0.05, **P < 0.01 compared with the immunized control (saline).

Activated TLR4 signalling induces oxidative stress, resulting in imbalance of antioxidant system^25^,^26^. Excessive production of pro-inflammatory mediators exaggerates the oxidative stress in RA, resulting in immune cells activation^27^. Since the two herbs in RL contain various natural antioxidants^21^,^28^,^29^, we determined the effects of RL on the activities of the endogenous antioxidant enzymes in CIA rats. RL treatment at high dose significantly rejuvenated the declined activities of the antioxidant enzymes (SOD, CAT and GSH-Px) in sera (Fig. 6A) and liver tissues of CIA rats (Fig. 6B).

### RL suppressed pathogenic immune responses in CIA rats

**Figure 7:**
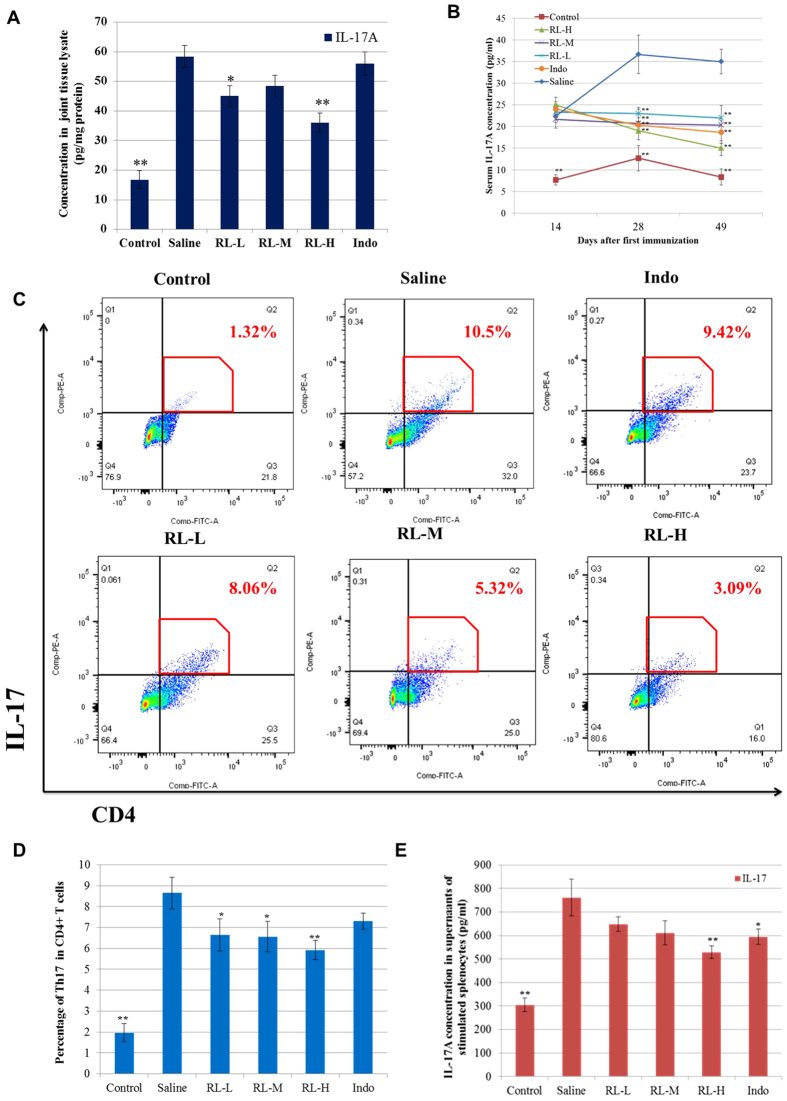
Effects of RL on IL-17 and Th17 cells in CIA rats. (**A**) Levels of IL-17A from joint tissue lysates determined by Milliplex MAP Rat Cytokine/Chemokine Panel at the end of the experiment. (**B**) Serum levels of IL-17 determined by Milliplex MAP Rat Cytokine/Chemokine Panel at different time points. (**C)** Isolated splenocytes were stimulated with PMA (0.05 μg/ml) and ionomycin (1 μg/ml) for 4 h and then BFA (5 μg/ml) for additional 2 h. Cells were extracellularly stained with FITC-conjugated anti-CD4, fixed, permeabilized and labelled with PE-conjugated anti-IL-17A. Representative graphs showing the percentages of positive-stained Th17 in CD4^+^ cells analyzed by flow cytometry. (**D**) The mean percentages of positive-stained Th17 in CD4^+^ cells. (**E**) Concentration of IL-17A determined by ELISA in the supernatants of isolated splenocytes stimulated with PMA (0.05 μg/ml) and ionomycin (1 μg/ml). Values are the mean ± SEM (n = 8). *P < 0.05, **P < 0.01 compared with the immunized control (saline).

**Figure 8:**
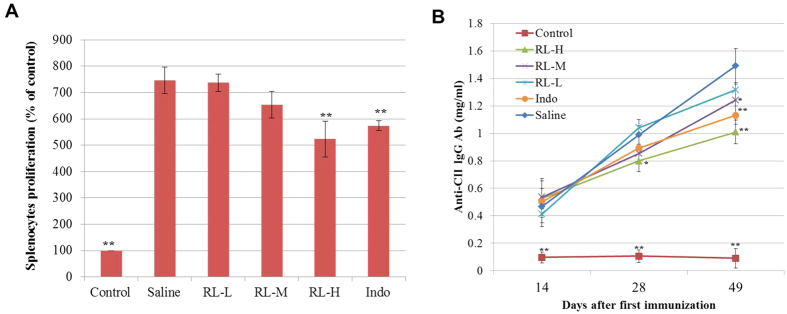
Effects of RL on immune responses in CIA rats. (**A**) Splenocytes isolated from rats were stimulated with CII (50 μg/ml) and cultured for 72 h. Cell proliferation was measured by the MTT assay and expressed as mean percentage of control cells. (**B**) Antibody concentrations in the sera were measured by ELISA. Values are the mean ± SEM (n = 8). *P < 0.05, **P < 0.01 compared with the immunized control (saline).

TLR4 potently instructs both innate and adaptive immune responses. Inhibition on TLR4 signalling has been shown to lower the synovial expression of IL-17 and serum concentrations of IL-17 in CIA models^6^,^30^. In order to characterize the effect of RL on TLR4-signalling-related immune responses, levels of IL-17 in joint tissues and sera were determined. RL significantly mitigated the levels of IL-17 in both sera and joint tissue lysates of CIA rats (Fig. 7A,B). Studies showed that the splenic proportion of pathogenic Th17 cells, which play an important role in the pathogenesis of RA^31^, correlated directly with the levels of serum IL-17 in arthritic mice^32^. In line with decreasing serum IL-17, RL also reduced Th17 cell proportion in splenocytes (Fig. 7C,D) and inhibited IL-17A production in PMA-and-ionomycin-activated splenocytes isolated from CIA rats (Fig. 7E). The humoral response against autoantigen is essential for the development of erosive arthritis^33^. RL inhibited CII-stimulated proliferation of splenocytes isolated from CIA rats (Fig. 8A) and significantly reduced the IgG antibody level in sera (Fig. 8B).

## Discussion

Current drug treatment options against RA like NSAID and disease-modifying anti-rheumatic drugs are not satisfactory because of their low efficacy, adverse effects and toxicity. New therapeutic agents with low toxicity and high efficacy should be explored. Researchers are seeking alternative, complementary therapeutic agents from multi-target Chinese medicines.

RL, a traditional remedy for inflammatory disorders including RA, is composed of *Yingshi* and *Jinyinhua,* which are of low toxicity. *Yingshi* can be used to make various food products^28^,^34^,^35^, and *Jinyinhua* is treated as food in Asian countries^36^. It has been shown that oral administration of an extract of *Yingshi* attenuated the severity of CIA rats without showing any observable adverse effects^21^. *Jinyinhua* together with other herbs have been demonstrated to improve conditions in CIA mice^37^. In the present study, we demonstrated that the herbal formula RL significantly improved the clinical conditions throughout the course of disease in CIA rats without observable adverse effects. These evidences support RL to be a potential safe and effective anti-RA agent.

**Figure 9:**
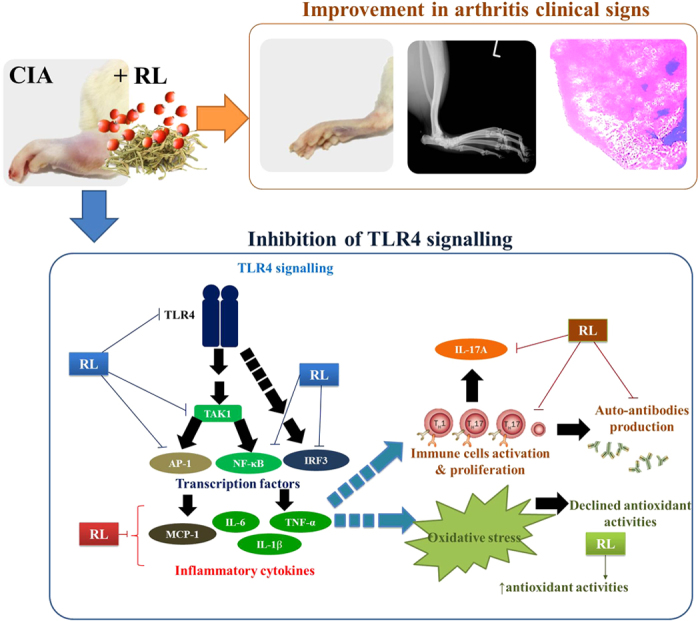
A schematic diagram showing the effects of RL in CIA rats.

Several studies have proposed novel strategies for the treatment of RA and CIA by regulating the activities of immune cells as well as the levels of certain cytokines (e.g. TNF-α, GMCSF, IL-17)^38^,^39^. The TLR4 signalling pathway, which regulates a wide range of proinflammatory cytokines and the activities of various immune cells, is believed to be one of the novel therapeutic targets in RA management^11^,^12^. We previously reported that RL could inhibit various TLR4 signalling components^23^. Therefore, we investigated RL’s effects on TLR4 signalling-related events in CIA rats, in which TLR4 can be activated by ligands from damaged synovial tissues^6^. Upon ligand binding, TLR4 signalling activates transcription factors NF-κB (p50/p65 heterodimer), AP-1 (c-Fos/c-Jun heterodimer) and IRF3^40^,^41^. Activation of these transcription factors results in the production of inflammatory mediators e.g. IL-6, MCP-1, TNF-α and IL-1β, providing a positive feedback to the TLR4 signalling pathways, which results in persistent inflammation and favours the induction of autoimmunity^42^. In this study, we found that RL could inhibit TLR4 signalling. This was demonstrated by the suppressed protein expressions of TLR4, phospho-TAK1, phospho-NF-κB p65, phospho-c-Jun, and phospho-IRF3 in joint tissues. Phosphorylation of the key transcription factors of TLR4 signalling is essential to the production of pro-inflammatory cytokines. RL’s inhibition on TLR4 signalling was also evidenced by the reduced production of pro-inflammatory cytokines regulated by the transcription factors NF-κB, AP-1 and IRF3 in sera and joint tissues of CIA rats. These pro-inflammatory cytokines could induce oxidative stress, leading to declined activities of endogenous antioxidants. RL rejuvenated the declined activities of the antioxidant enzymes (SOD, CAT and GSH-Px) in sera and liver tissues of CIA rats. TLR4 signalling also takes part in immune responses^43^. Production of autoimmune antibodies and IL-17 upon collagen immunization was found to be less in TLR4-deficient mice than in wild type mice^6^. In the present study, RL was found to be able to lower the levels of IL-17 in sera and joint tissues of CIA rats. This observation was supported by the reduction of pathogenic Th17 cells and IL-17 production in spleen cells. In addition, RL could reduce the IgG antibody production and inhibit antigen-stimulated proliferation of splenocytes. These suggest that attenuation of TLR4 signalling is possibly one of the molecular mechanisms for the anti-arthritic effects of RL in CIA rats (Fig. 9). Reduction in the proportions of Th17 cells may also be responsible for the effects of RL. Several studies have demonstrated that besides the Th17 cells, modulating the activities of other immune cells (e.g. macrophages, monocytes, B cells, dendritic cells, Treg cells) may also be a potential therapeutic approach in RA management^44^,^45^,^46^,^47^. Because of the multi-component and multi-target natures of Chinese medicines, we believe that this herbal formula may also affect other immune cells. Further studies are needed to investigate the effects of this formula on other signalling pathways as well as other components of the immune system.

In conclusion, RL improved the clinical conditions of CIA rats without overt adverse effects, which was associated with the inhibition of TLR4 signalling. These findings provide further pharmacological basis for the traditional use of this formula in controlling RA. Further studies should be performed to develop RL into a safe, effective and modern anti-RA agent.

## Methods

### Preparation of RL

The herbal material was prepared as described previously^23^. Briefly, 500 g *Yingshi* and 300 g *Jinyinhua* were mixed, minced, macerated with 12 L absolute ethanol for 24 h, and refluxed for 2 h twice. Supernatants were filtered, concentrated, freeze-dried and produced RL (Yield: 15.2%). To control its quality, we established an HPLC chromatogram for RL and quantified the contents of gallic acid and chlorogenic acid (Supplementary Figure 1), which is the same as the Supplementary Material of a previous report^23^.

### Animal treatments

Male Wistar rats (140 ± 15 g) were supplied by the Chinese University of Hong Kong and housed under standard conditions (25 ± 2 °C, humidity: 60 ± 10%, 12 h-light:12 h-dark) with free access to water and chow. Forty-eight rats were randomly divided into six groups of eight. Five groups were immunized intradermally, at the base of the tail and the back, with 200 μg bovine type II collagen (Chondrex, Redmond, WA, USA) in 0.05 M acetic acid emulsified with equal volume incomplete Freund’s adjuvant (IFA) (Chondrex, Redmond, WA, USA) on day 0. A boost injection of 100 μg collagen-IFA suspension was given in the same manner on day 7. From day 14 to 56, immunized groups were intragastrically (i.g.) administered with 165, 330, 660 mg RL/kg/day, 2.5 mg indomethacin/kg/day and saline, respectively; while normal controls were i.g. administered with saline. Retro-orbital blood samplings under anaesthesia were performed at various time points. Sera were collected by centrifugation (1500 G, 20 min). At day 57, the rats were fasted overnight and killed by anaesthetic overdose. After taking radiographs of hind limbs, left paw tissues (whole joints including synovium, adjacent tissues and bones) were pulverized using a mortar and pestle filled with liquid nitrogen, and homogenized in 50 mM Tris-HCl, 1% NP-40, 0.35% sodium deoxycholate, 150 mM NaCl, 1 mM EDTA of pH 7.4, 1 mM phenylmethylsulfonyl fluoride, 1 mM NaF, 1 mM Na_3_VO_4_, 10 μg/mL aprotinin, 10 μg/mL leupetin and 10 μg/mL pepstatin A. Right hind limbs were collected for histological examinations. Single cell suspensions of splenocytes were prepared from spleens as described^6^. Red blood cells in the suspensions were removed by treatment with 0.16 M Tris-NH_4_Cl solution. Liver tissues were dissected and homogenized in ice-cold saline using an ultra-Turrax T-25 homogenizer. The experimental protocol was approved by the Ethics Committee of Hong Kong Baptist University. All experimental procedures were conducted according to the principles expressed in the Declaration of Helsinki and the Guide for the Care and Use of Laboratory Animals published by the US National Institutes of Health. Every effort was made to reduce the number of animals used and minimize their pain and distress.

### Macroscopic scoring of CIA

Each hind limb was scored on a 0 ~ 4 scale: 0 = normal; 1 = mild, but definite ankle erythema and swelling, or limited to individual digits; 2 = moderate ankle erythema and swelling; 3 = entire limb erythema and swelling including digits; 4 = maximally inflamed limb with multiple joints involvement. The arthritis score was the sum of the scores of the two hind limbs (maximum: 8). To quantify oedema, hind paw volumes were measured using a plethysmometer.

### Radiographic analysis

Hind limbs radiographs taken on day 57 were evaluated using a 0 ~ 3 scale (maximum: 6): 0 = normal; 1 = soft tissue swelling only; 2 = soft tissue swelling and early joint erosions; 3 = severe bone erosion or significant osteophyte formation.

### Histological analysis

Right hind limbs were fixed in 4% neutral formalin for one week and then decalcified in 0.5 M EDTA and 0.5% paraformaldehyde for 20 days. Decalcified samples were dehydrated with alcohol, embedded in paraffin and sectioned (10 μ thick). Talocrural joint sections were stained with hematoxylin and eosin and mounted on glass slides for histological analysis by light microscopy. Histopathological changes of the joint were evaluated based on histologic parameters (inflammation, synovial hyperplasia, pannus formation, and erosion of cartilage/bone) using a 0 ~ 4-point scale: 0 = normal; 1 = synovium hypertrophy with cell infiltrates; 2 = pannus and cartilage erosions; 3 = major cartilage and subchondral bone erosions; 4 = loss of joint integrity and ankylosis.

### Immunoblotting

The quantified joint tissue lysates were assessed by Western blot analysis as described previously^23^. Briefly, protein lysates were subjected to 10% SDS-PAGE and then electro-transferred onto nitrocellulose membrane. After blocking with 5% non-fat milk in TBST, the membrane was incubated with designated primary antibodies overnight. The membrane was then incubated with anti-mouse or anti-rabbit secondary antibodies^48^,^49^. The specific immunoreactive bands were detected using enhanced chemiluminescence ECL detection kit (Invitrogen, Carlsbad, CA, USA) following the manufacturer’s instruction.

### Splenocytes culture

Splenocytes isolated from animals were maintained in RPMI 1640 medium containing 10% heat-inactivated foetal bovine serum (FBS) and 1% antibiotics of penicillin/ streptomycin at 37 °C under 5% CO_2_.

### Splenocytes proliferation assay

Splenocytes suspensions (5 × 10^6^ cells/well in 96 well plate) were cultured in RPMI 1640 medium containing 50 μg/ml heat-denatured bovine CII (pre-treated at 80 °C, 10 min) for 72 h. MTT (0.5 mg/ml) was then added for 1 h. Supernatants were removed after centrifugation (500G, 10 min), and 100 μl of DMSO were added. Absorbance at 570 nm, measured using microplate spectrophotometer, was expressed as percentage of the normal control.

### Splenocytes activation assay

Splenocytes (2 × 10^6^ cells/ml) were stimulated with PMA (0.05 μg/ml) and ionomycin (1 μg/ml) at 37 °C for 48 h. IL-17A concentrations in the supernatants were determined by ELISA (eBioscience, Inc., CA, USA) following manufacturer’s instructions.

### Flow cytometric analysis

Splenocytes (2 × 10^6^ cells/ml) were stimulated with PMA (0.05 μg/ml) and ionomycin (1 μg/ml) for 4 h and then brefeldin A (BFA, 5 μg/ml) for 2 h. Cells were extracellularly stained with FITC-conjugated anti-CD4, fixed, permeabilized, then labelled intracellularly with PE-conjugated anti-IL-17A^50^,^51^. Percentages of stained cells were measured using FACS instruments.

### Cytokine, chemokine, antioxidant and anti-collagen antibody assays

Cytokine and chemokine levels were quantified by Milliplex MAP Rat Cytokine/Chemokine Panel (Merck Millipore), while antioxidants and anti-mouse CII IgG levels were determined by ELISA kits (eBioscience, Inc., CA, USA) following manufacturer’s instructions^52^.

### Statistical analysis

The results are presented as the means ± SEM. Data were analyzed by one-way ANOVA. Comparisons between two groups were performed using the Dunnett’s multiple comparisons test or post-hoc analysis. Statistical analyses were carried out using GraphPad Prism version 5.0 (GraphPad Software, San Diego, CA, USA). *P* < 0.05 was considered statistically significant.

## Additional Information

**How to cite this article**: Cheng, B. C. Y. *et al*. A herbal formula comprising Rosae Multiflorae Fructus and Lonicerae Japonicae Flos, attenuates collagen-induced arthritis and inhibits TLR4 signalling in rats. *Sci. Rep.* **6**, 20042; doi: 10.1038/srep20042 (2016).

## Supplementary Material

Supplementary Information
