# Supplementary material for: A herbal formula comprising Rosae Multiflorae Fructus and Lonicerae Japonicae Flos, attenuates collagen-induced arthritis and inhibits TLR4 signalling in rats
Source: Sci Rep. 2016 Feb 10;6:20042. doi: 10.1038/srep20042 (PMC4748217; doi:10.1038/srep20042)
Supplement: Supplementary Information [file srep20042-s1.pdf]

A herbal formula comprising Rosae Multiflorae Fructus and Lonicerae Japonicae Flos, attenuates collagen-induced arthritis and inhibits TLR4 signalling in rats

Authors

Brian Chi Yan CHENG[1,2,3], Hua YU[1,2,3], Hui GUO[1,2,3], Tao SU[1,2,3], Xiu-Qiong FU[1,2,3], Ting LI[1,2,3], Hui-Hui CAO[1,2,3], Anfernee Kai-Wing TSE[1,2,3], Zheng-Zhi Wu[4], Hiu-Yee KWAN[1,2,3], Zhi-Ling YU[1,2,3,*]

Affiliations

[1] Consun Chinese Medicines Research Centre for Renal Diseases, Hong Kong Baptist University, Hong Kong

[2] Centre for Cancer and Inflammation Research, School of Chinese Medicine, Hong Kong Baptist University, Hong Kong

[3] Institute of Integrated Bioinfomedicine & Translational Science, HKBU Shenzhen Research Institute and Continuing Education, Shenzhen, PRC

[4] Shenzhen Institute of Geriatrics, Shenzhen, PRC

* Corresponding author: School of Chinese Medicine, Hong Kong Baptist University, Hong Kong. Tel: +852 34112465; Fax: +852 34112461; E-mail: zlyu@hkbu.edu.hk

Supplementary figure

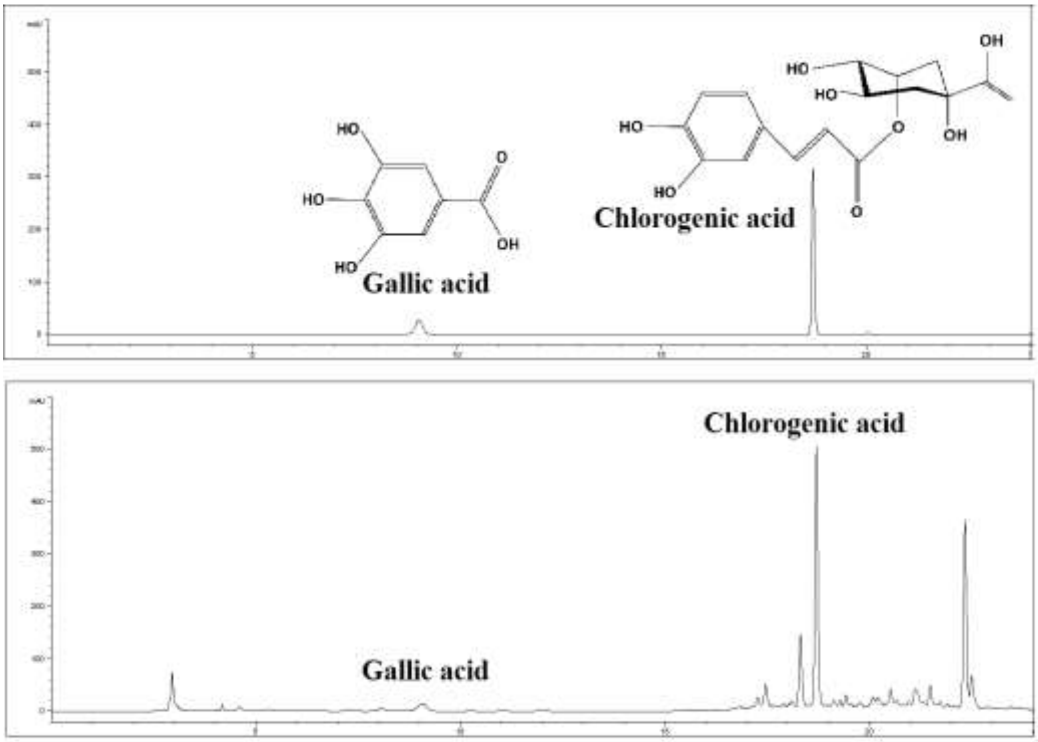

Supplementary Figure 1. High performance liquid chromatography chromatograms of the standards (gallic acid and chlorogenic acid, upper panel), and RL (lower panel) detected at 272 nm. The mean content of gallic acid per gram of RL was 763 μg, while that of chlorogenic acid was 26,399 μg. The results were presented previously[23].
